# Additively Manufactured Detection Module with Integrated Tuning Fork for Enhanced Photo-Acoustic Spectroscopy

**DOI:** 10.3390/s22197193

**Published:** 2022-09-22

**Authors:** Roberto Viola, Nicola Liberatore, Sandro Mengali

**Affiliations:** Consorzio CREO, 67100 L’Aquila, Italy

**Keywords:** gas analyzer, PAS, tuning fork, micro additive manufacturing, interferometric readout

## Abstract

Starting from Quartz-Enhanced Photo-Acoustic Spectroscopy (QEPAS), we have explored the potential of a tightly linked method of gas/vapor sensing, from now on referred to as Tuning-Fork-Enhanced Photo-Acoustic Spectroscopy (TFEPAS). TFEPAS utilizes a non-piezoelectric metal or dielectric tuning fork to transduce the photoacoustic excitation and an optical interferometric readout to measure the amplitude of the tuning fork vibration. In particular, we have devised a solution based on Additive Manufacturing (AM) for the Absorption Detection Module (ADM). The novelty of our solution is that the ADM is entirely built monolithically by Micro-Metal Laser Sintering (MMLS) or other AM techniques to achieve easier and more cost-effective customization, extreme miniaturization of internal volumes, automatic alignment of the tuning fork with the acoustic micro-resonators, and operation at high temperature. This paper reports on preliminary experimental results achieved with ammonia at parts-per-million concentration in nitrogen to demonstrate the feasibility of the proposed solution. Prospectively, the proposed TFEPAS solution appears particularly suited for hyphenation to micro-Gas Chromatography and for the analysis of complex solid and liquid traces samples, including compounds with low volatility such as illicit drugs, explosives, and persistent chemical warfare agents.

## 1. Introduction

QEPAS [1,2] has emerged in the last two decades as one of the most promising spectroscopies for the sensing of gases and vapors [3,4]. The technique has been tested successfully with a variety of compounds [5,6,7,8,9] and proven to achieve excellent sensitivity [10,11] and selectivity [12,13]. More recently, QEPAS has been hyphenated to micro-Gas Chromatography for the analysis of complex vapor traces [14]. However, there are technical issues that can limit the use of a QEPAS sensor and affect its performance. First, the integration of the ADM requires accurate manual positioning and alignment of its components, particularly if one wants to include acoustic micro-resonators to improve sensitivity. Secondly, the customization of the quartz tuning fork for specific applications requires a dedicated design and micro-machining process [15], which may be complex and expensive. Finally, when using commercial tuning forks, one has to either remove the welds or work at temperatures below the melting point of the welds. Operating room temperature is a severe limitation when dealing with low volatility and sticky compounds, which tend to condense inside the ADM, causing sensor contamination, decreased sensitivity, and memory effects [16]. Unlike QEPAS, TFEPAS is based on a non-piezoelectric tuning fork and an interferometric optical read-out, which has already been proven effective at measuring the photoacoustic excitation of a quartz tuning fork [17]. We present a miniaturized photoacoustic device for TFEPAS, in which the novelty is that the fork, acoustic micro-resonator, and ADM body are built monolithically into one block using MMLS or other AM techniques. This is a remarkable difference from QEPAS, in which AM techniques are used only to build the ADM body [18], and also from other TFEPAS [17]. The wider use of AM in TFEPAS has several advantages. First, there is easier customization of the shape and size of the tuning fork by translating design changes into changes to the Standard for the Exchange of Product Data (STEP) file for the AM machine. Second is the elimination of manual operations for fine positioning and alignment of the fork and resonators. Third, there is greater miniaturization of the external and internal volumes of the ADM, with inner walls that can be shaped around the tuning fork without the need to ensure manual accessibility. Finally, the absence of welds for electric contacts and the elimination of coefficient of thermal expansion differentials between the various parts and materials of the ADM allows for increased operating temperature and, consequently, the elimination of misalignments induced by high temperature.

## 2. Materials and Methods

### 2.1. Additively Manufactured ADM

**Figure 1:**
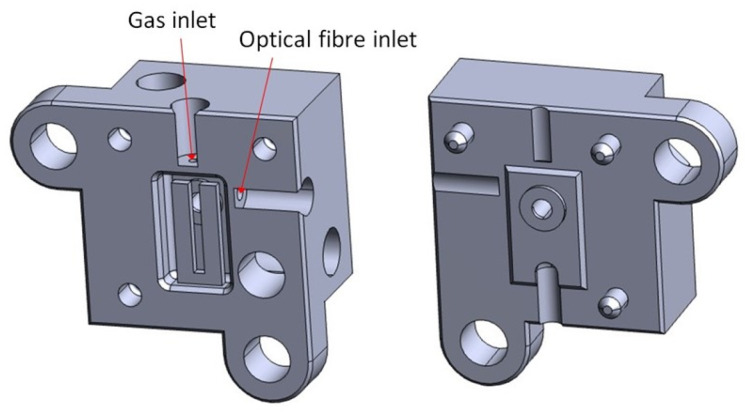
STEP models for additive manufacturing of the two monolithic stainless-steel parts of the TFEPAS ADM. The red arrows indicate two small apertures, giving access to the gas sample for analysis and to the optical fiber for interferometric readout.

To explore the feasibility of the TFEPAS solution, we designed an ADM in which the tuning fork, the acoustic micro-resonator, and the body of the surrounding analysis cell are integrated in a compact monolithic piece of stainless steel, which is a material with good corrosion resistance, also suitable for manufacturing by MMLS. The actual design, which is illustrated in Figure 1, involves the construction of two distinct parts to be assembled together. This allows visual inspection of the fork and easy interfacing of the ADM with the microfluidic system and the interferometric readout.

**Figure 2:**
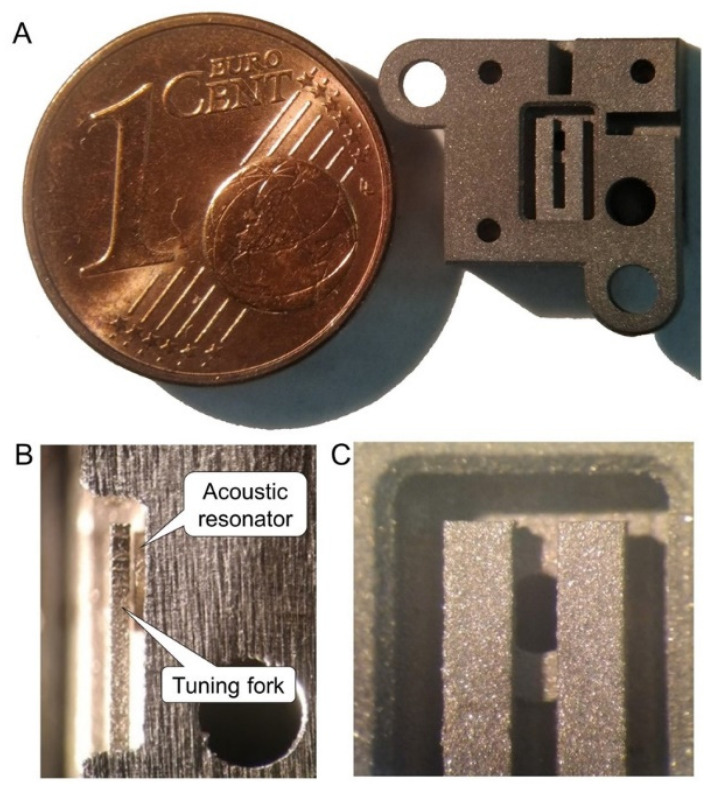
(**A**) Picture of the AM part of the ADM, and size comparison with a 1 cent coin. (**B**) Detail of a lateral view of the tuning fork close to contact with the micro-acoustic resonator. (**C**) front view of the tuning fork with micro-resonator behind, showing the roughness of AM surfaces.

Given the small size and high spatial resolution required for the tuning fork and the acoustic micro-resonator, we started by verifying the feasibility by MMLS of geometries with sub-millimeter details. We designed our tuning fork (TF) a bit larger than a standard quartz tuning fork (QTF) to facilitate beam focusing but to avoid decreasing the photoacoustic pressure on the prongs too much. Moreover, we want to have a resonant frequency around 30 kHz, similar to a standard QTF, and a small internal volume of the cell for analysis. We have designed a tuning fork that has tines that are 4 mm long, 0.75 mm wide, 0.4 mm thick, and separated by a 0.5 mm gap and a micro-resonator with an internal diameter of 0.9 mm positioned at 0.1 mm from the fork. Next, to contain costs, we commissioned Precipart, a Swiss company, to manufacture only the main two parts of the ADM, i.e., the one with the tuning fork (on the left in Figure 1). From visual microscope inspection, the laser-sintered part matches the STEP model of Figure 1 very well, as shown in Figure 2A, where the piece is placed next to one euro cent as a size indicator. To better inspect the final position of the fork in respect to the resonator, one sample of the ADM part was cut close to the lateral surface of the fork. A detail of the cut section is shown in Figure 2B. It can be seen how the fork is positioned very close to the acoustic micro-resonator without touching it, as required to allow vibration with minimal damping and, at the same time, to realize effective photoacoustic coupling.

### 2.2. Optical Readout

**Figure 3:**
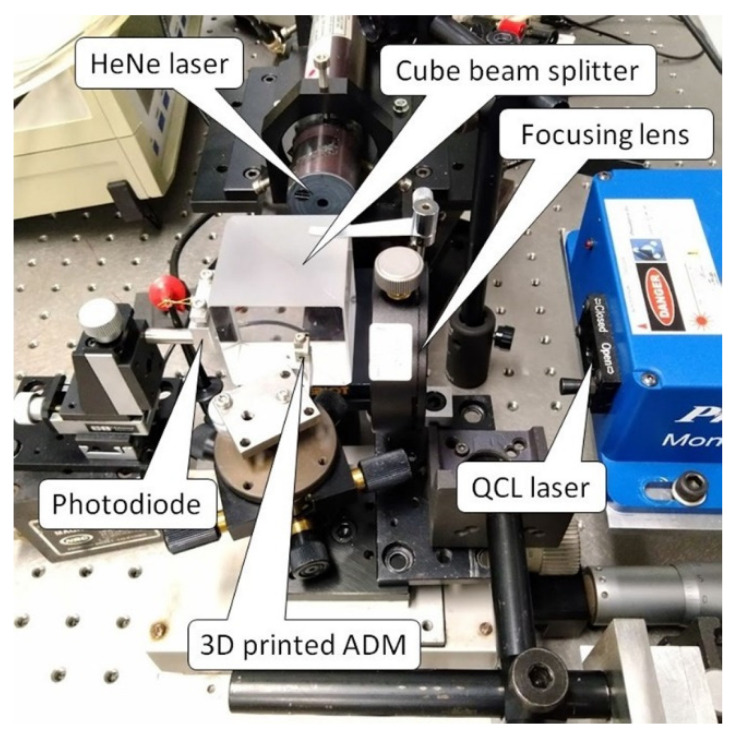
Picture of the test bench for interferometric readout of the micro-3D-printed tuning fork.

**Figure 4:**
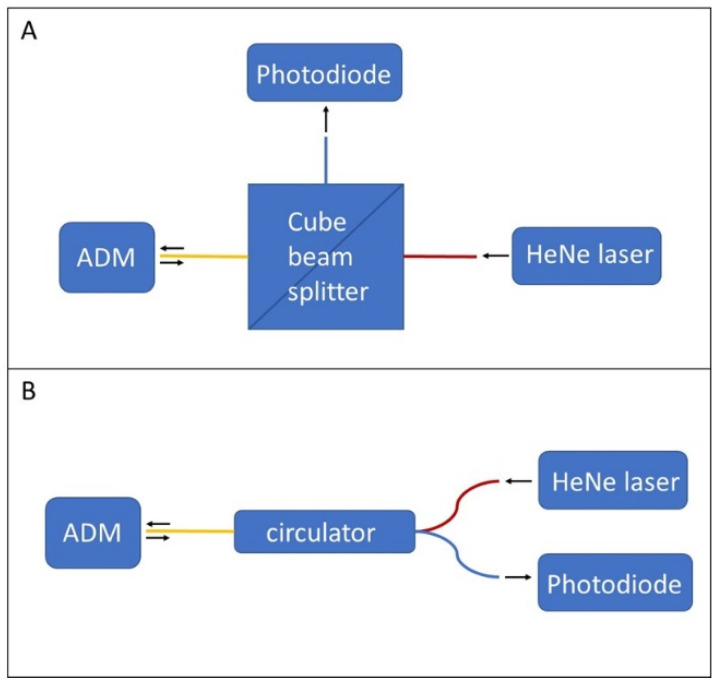
Optical schemes for the interferometric readout system. (**A**): free space solution based on a cube beam splitter; (**B**): more embedded solution based on optical fiber circulator.

The interferometric readout has already been proved to give results comparable to a piezoelectric readout in terms of both sensitivity and signal-to-noise ratio [19]. In this study, the feasibility of the interferometric readout was verified on an AM stainless-steel tuning fork. While a flat polished surface is desirable to maximize reflection from the metallic tuning fork, the surface finish resulting from the MMLS process appears rough and opaque. This can be seen in Figure 2C, which shows details of the ADM with a portion of the fork prongs in front of the circular aperture of the acoustic micro-resonator. The actual roughness of the AM surfaces measured by profilometry was found to have an average value of approximately 1.5 microns, with peak-to-peak values even greater than 10 microns. We have solved the problem with mechanical polishing of the surface of the fork where the optical interrogation takes place. Prospectively, mechanical polishing shall be replaced by wet or plasma polishing. A test bench, which is depicted in Figure 3, was set up to verify the functionality of the AM tuning fork as a resonant element and demonstrated the feasibility of its vibration amplitude measurement by interferometric readout. The set up essentially consists of one HeNe laser (Uniphase, model: 1103P), one photodiode (OSI Optoelectronics, model: OSD1-0), one cube beam splitter, the AM part of the ADM, plus supports and micro-manipulators for precision positioning. The components are arranged into a Michelson interferometer configuration, as shown in Figure 4A, to interrogate the lateral surface of the tuning fork. A Quantum Cascade Laser (QCL Monolux from Pranalytica) is used to stimulate the tuning fork vibration. Vibration can be induced by photothermal excitation, i.e., by focusing laser pulses directly on the fork. Alternatively, it can be induced by photoacoustic excitation as in a QEPAS sensor, i.e., by focusing the laser pulses on the gap between the prongs in the presence of a gas that absorbs the laser radiation. In both cases, the interferometric readout signal is obtained by extracting the component of the photodiode signal at the same modulation frequency of the QCL, by means of a spectrum analyzer (Stanford Research Systems, model: SR780).

**Figure 5:**
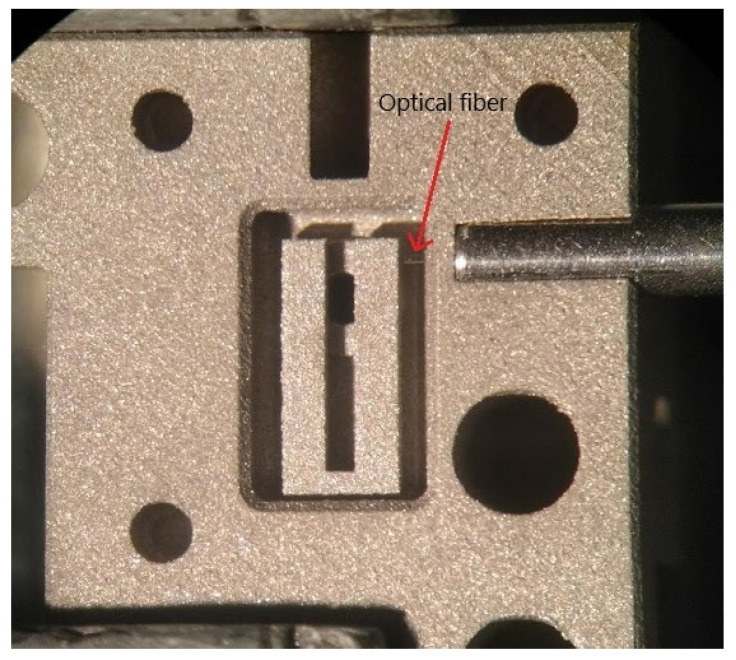
Picture of the AM part of the ADM showing the end ferrule of the optical fiber for interferometric readout locked to the ADM. The red arrow indicates the optical fiber protruding to get close to the tuning fork.

In our intentions, the TFEPAS sensor should be robust, portable, and fieldable. Therefore, with regard to the interferometric readout, a second configuration was also considered, in which free space coupling (Figure 4A) is replaced by optical fiber coupling (Figure 4B) by means of a fiber optic circulator (Thorlabs model: WMC3L1F). In this new configuration, the HeNe laser beam is focused on the input port of the optical circulator and travels unattenuated towards the ADM. The interferometric signal from the ADM travels back unattenuated through the circulator and is acquired by the photodiode, while back reflections towards the laser are strongly attenuated. The optical fiber of the circulator can be inserted and locked to the ADM to maximize versatility and robustness and to avoid misalignment problems. For this purpose, on one side of the ADM there is a pilot hole for the insertion of the optical fiber, which allows the interferometric readout of the tuning fork vibration throughout a diaphragm of 0.5 mm in diameter (the optical fiber inlet shown in the STEP model of Figure 1). In Figure 5, the ferrule of one termination of the fiber inserted on the AM part of the ADM is shown. The optical fiber, as indicated by the red arrow in Figure 5, is protruding from the ferrule end stop, across the diaphragms, to get close to the lateral face of the fork and improve the collection of laser radiation back-reflected by the fork.

## 3. Results and Discussion

### 3.1. Resonant Frequency and Q Factor

**Figure 6:**
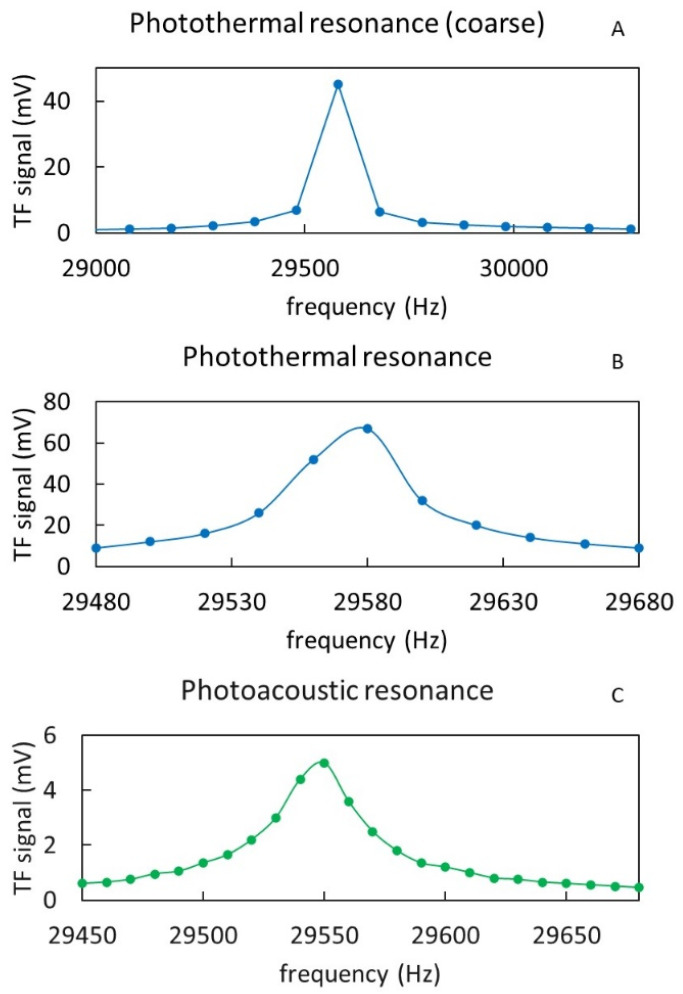
Response of the AM tuning fork to coarse- (**A**) and fine-tuning (**B**) of the photothermal excitation around the resonance frequency. Similar response to photoacoustic excitation frequency around the resonance frequency (**C**).

A first characterization of the AM tuning fork resonance is made by focusing the QCL laser directly at the base of the fork to induce photo-thermal excitation. The emission of the laser at a fixed wavelength is modulated externally. While varying the frequency of modulation, the component of the interferometric readout signal at the same frequency is acquired through a spectrum analyzer. Figure 6A shows a coarse scan of the modulation frequency searching for the peak of resonance, while Figure 6B shows a refined scan around the resonance peak. From the result shown in Figure 6B, a resonance peak at 29,580 Hz with a Q factor of ca. 700 can be estimated. For comparison, a standard QTF has a resonant peak at ca. 32,758 Hz in the air, with a Q factor in the range of 1000–10,000, depending on the configuration of the acoustic micro-resonator [20]. In another test, similar to the previous one, the QCL laser was instead focused on the gap between the two prongs of the fork (as for QEPAS) to induce photo-acoustic excitation, in the presence of a flow of ammonia at 100 ppm in nitrogen. In this case, the QCL emission is modulated at fixed wavelength of 9218 nm, corresponding to an absorption peak of ammonia. As for previous tests, the component of the interferometric signal at the modulation frequency of the QCL is plotted in Figure 6C around the fork resonance. A similar resonance peak at 29,550 Hz and a Q factor of about 700 are also measured in this case. Note that starting from the theoretical formula for the resonance frequency *f*_0_ of the tuning fork (fundamental harmonic mode) [21], which is expressed in Equation (1):

(1) $$f_{0}=\frac{\pi T}{8\sqrt{12}L^{2}}\sqrt{\frac{E}{\rho }}$$

where *ρ* is the density of the fork material, *E* is the Young modulus, *T* is the prong thickness, *L* is the prong length, and entering the design parameters of the metallic fork, one obtains *f*_0_ ≈ 37 KHz. Differences between the theory and the experiment can be explained assuming minor deviations from the design parameters, and, in particular, slightly different density and Young’s modulus for AM stainless steel with respect to bulk material and some thinning of the fork (e.g., decreasing *T*) deriving from the mechanical polishing process.

### 3.2. Sensitivity and Linearity of the Response

**Figure 7:**
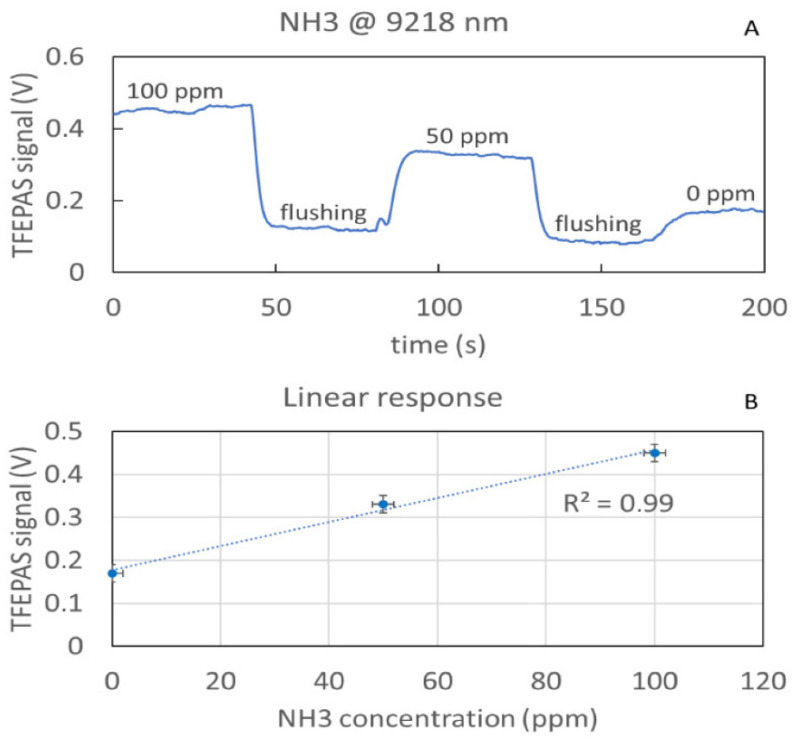
Real time measurement of the TFEPAS signal acquired while flushing different concentrations of ammonia (**A**) and corresponding scatter plot showing the linearity of the response (**B**).

A series of tests with ammonia at different concentrations were carried out to verify the performance obtainable with the AM TFEPAS device in terms of sensitivity and linearity of the response. For these tests, a cylinder containing a sample mixture of 100 ppm of ammonia (NH3) in nitrogen was used. The cylinder was connected to a gas box equipped with mass-flow controllers, which allows the adjustment of the flow and to reduce the concentration of ammonia delivered by adding a make-up flow of nitrogen. The plot in Figure 7A shows the photoacoustic signal measured by interferometric readout, having the QCL tuned at the ammonia absorption peak at 9218 nm, while the concentration of ammonia is varying. The flux was set to 50 sccm while measuring the mix samples and to 500 sccm for flushing. This higher flux forced the fork to vibration unlinked to the photoacoustic excitation. The corresponding scatter plot in Figure 7B shows good linearity between response and concentration. The interferometric signal was acquired by connecting the reading photodiode to a current preamplifier (Stanford Research Systems, model: SR570) and the latter to a lock-in amplifier (EG&G Princeton Applied Research, model 5210) synchronized to the QCL signal modulated at the experimentally measured resonance frequency of the tuning fork of 29,550 Hz and a lock-in time constant of 1 s. From the analysis of the signal-to-noise ratio (S/N), a limit of detection (LOD) better than 1 ppm is estimated. Note that the current AM part of the TFEPAS ADM implements only one half of the acoustic micro-resonator, and therefore, an even higher sensitivity could be achieved with the full ADM.

### 3.3. Spectral Resolution

**Figure 8:**
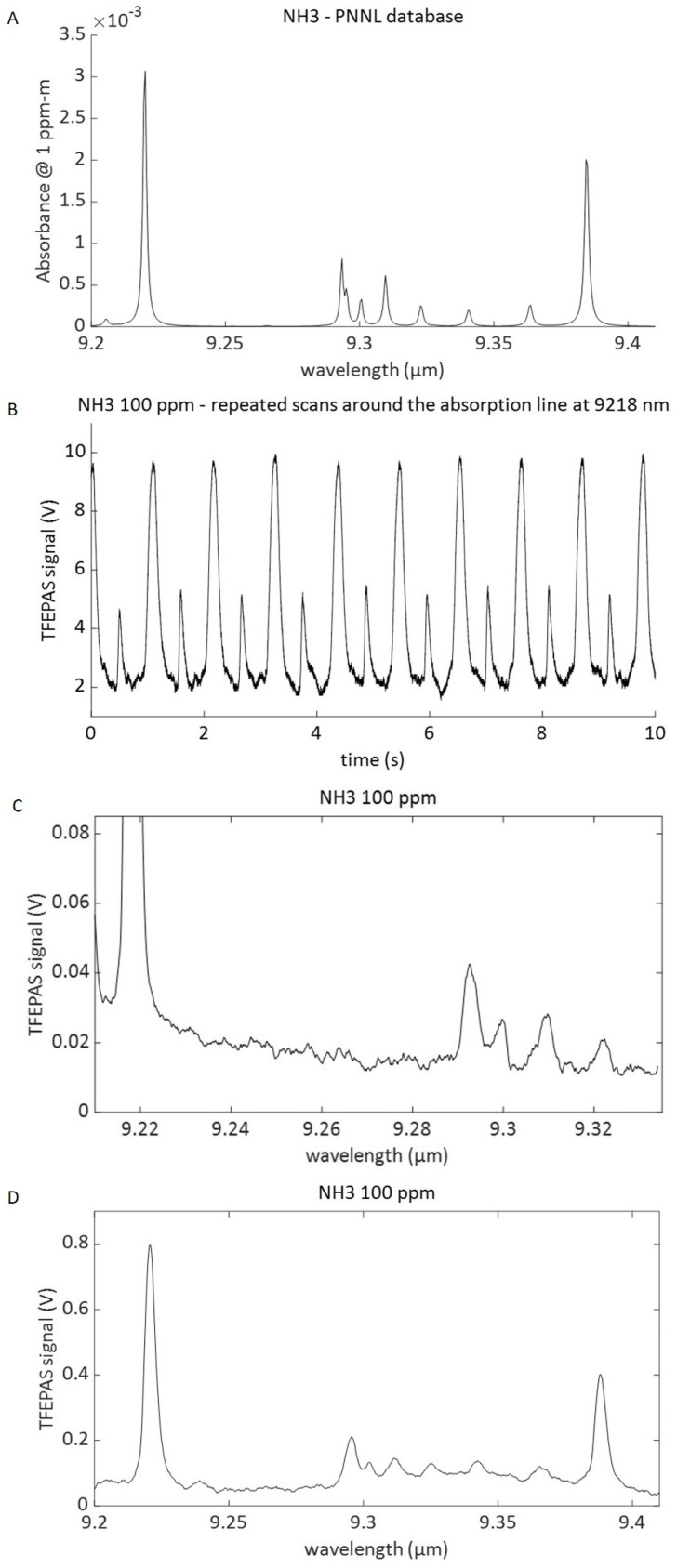
(**A**) Reference IR absorption spectrum of ammonia from PNNL database. (**B**) Real-time acquisition of the signal from 100 ppm of ammonia while cyclically scanning the emission wavelength of the QCL across the IR absorption line at 9218 nm. (**C**) Absorption spectrum of ammonia measured in the wavelength spectral range 9210–9335 nm. The strongest peak at 9218 nm is out of scale for showing all the other absorption peaks detected better. (**D**) Spectrum measured in the same wavelength spectral range of the reference (9200–9410 nm).

The same gas model of ammonia 100 ppm in nitrogen was also used for testing the spectral resolution of the AM TFEPAS device over a broad spectral range in order to evaluate its capability to identify infrared (IR) absorption spectra. In these tests, the QCL laser scans several IR absorption lines of ammonia. The reference infrared absorption spectrum of ammonia, corresponding to the spectral range considered, was retrieved from the PNNL (Pacific Northwest National Laboratory) database [22] and is plotted in Figure 8A. The plot in Figure 8B shows the real-time acquisition of the interferometric TFEPAS signal measured during the cyclic QCL scan across the absorption line of ammonia at 9218 nm, in the interval 9210–9234 nm, using a lock-in time constant of 30 ms. Higher peaks correspond to forward scans and have an S/N of approx. 100, as in fixed wavelength tests, since the scan is slow enough compared to the fork response time. Conversely, lower peaks correspond to fast backward scans. The plot of Figure 8C refers to a wavelength scan in the range 9210–9335 nm, in which there are several absorption peaks of ammonia, as reported in Figure 8A. The most intense peak at 9218 nm is out of scale in this plot to enable a better definition of the weaker peaks from 9290 nm to 9330 nm. Comparison to the PNNL reference spectrum of Figure 8A shows a good match for all the detected peaks. Moreover, the TFEPAS sensor is able to resolve even weaker peaks of ammonia at 100 ppm, ensuring excellent identification of the substance at this concentration. The interferometric TFEPAS signal was acquired in this case using a lock-in time constant of 10 ms, and the total duration of the scan was 4 s. The plot in Figure 8D refers to an even wider scan of 9200–9410 nm. In this spectral range, there are eight absorption peaks of different strengths, all correctly resolved by the TFEPAS sensor, with an S/N around 10 for the less intense peaks and around 250 for the most intense peak at 9218 nm, which corresponds to a limit of detection of 400 ppb. In this case, the total duration of the scan was 20 s, and the lock-in time constant was 100 ms, to allow higher sensitivity at the cost of a longer scan duration. Assuming the absorption cross section of 1 × 10^−8^ cm^2^/mol for ammonia at 9218 nm, and the measured average laser power of 40 mW at the same wavelength, we estimate an NNEA of 3.4 × 10^−7^ cm^−1^ W Hz^−1/2^, which is quite modest in respect to the QEPAS state-of-the-art status, but can be probably be due to the lower Q factor of our metallic AM TF and the absence of the other half-acoustic micro-resonator.

## 4. Conclusions and Outlook

A photoacoustic solution, derived from the QEPAS sensing scheme, has been developed and tested. It is based on a metal AM monolithic ADM, which comprises a tuning fork plus an acoustic micro-resonator and is coupled with an interferential optical readout for transducing the photoacoustic excitation of the AM tuning fork into an electric signal. A TFEPAS ADM breadboard built in stainless steel by MMLS and connected to a bench-top interferometric readout has been tested successfully with ammonia, demonstrating an LOD better than 1 ppm, together with excellent identification potential, typical of PAS systems, deriving from the accurate measurement of IR fingerprints. Though showing a weaker performance in respect to the recorded sensitivity of QEPAS, the proposed solution overcomes some weak points of QEPAS, such as critical assembly and alignment of the tuning fork and the acoustic micro-resonators, difficulties in miniaturizing the internal volume of the ADM and operating it at high temperatures, and high costs for customizing the piezo tuning fork to specific applications. These features appear particularly interesting to extend the range of substances detectable by QEPAS, by adding sticky compounds with a high boiling point. As such, the solution presented paves the way for a new family of portable sensors for the identification of multi-component liquid and solid traces, in which TFEPAS analysis is hyphenated to micro-Gas Chromatographic chemical separation.

## References

[1] Kosterev A.A., Bakhirkin Y.A., Curl R.F., Tittel F.K. (2002). Quartz-enhanced photoacoustic spectroscopy. Opt. Lett..

[2] Kosterev A.A., Tittel F.K., Serebryakov D.V., Malinovsky A.L., Morozov I.V. (2005). Applications of quartz tuning forks in spectroscopic gas sensing. Rev. Sci. Instrum..

[3] Patimisco P., Scamarcio G., Tittel F.K., Spagnolo V. (2014). Quartz-enhanced photoacoustic spectroscopy: A review. Sensors.

[4] Ma Y. (2018). Review of Recent Advances in QEPAS-Based Trace Gas Sensing. Appl. Sci..

[5] Phillips M.C., Myers T.L., Wojcik M.D., Cannon B.D. (2007). External cavity quantum cascade laser for quartz tuning fork photoacoustic spectroscopy of broad absorption features. Opt. Lett..

[6] Bauer C., Willer U., Lewicki R., Pohlkötter A., Kosterev A., Kosynkin D., Tittel F.K., Schade W. (2009). A mid-infrared QEPAS sensor device for TATP detection. J. Phys. Conf. Ser..

[7] Pohlkötter A., Köhring M., Willer U., Schade W. (2010). Detection of molecular oxygen at low concentrations using quartz enhanced photoacoustic spectroscopy. Sensors.

[8] Waclawek J.P., Lewicki R., Moser H., Brandstetter M., Tittel F.K., Lendl B. (2014). Quartz-enhanced photoacoustic spectroscopy-based sensor system for sulfur dioxide detection using a CW DFB-QCL. Appl. Phys. B.

[9] Breitegger P., Schweighofer B., Wegleiter H., Knoll M., Lang B., Bergmann A. (2020). Towards low-cost QEPAS sensors for nitrogen dioxide detection. Photoacoustics.

[10] Spagnolo V., Patimisco P., Borri S., Scamarcio G., Bernacki B.E., Kriesel J. (2012). Part-per-trillion level SF6 detection using a quartz enhanced photoacoustic spectroscopy-based sensor with single-mode fiber-coupled quantum cascade laser excitation. Opt. Lett..

[11] Siciliani de Cumis M., Viciani S., Borri S., Patimisco P., Sampaolo A., Scamarcio G., De Natale P., D’Amato F., Spagnolo V. (2014). Widely-tunable mid-infrared fiber-coupled quartz-enhanced photoacoustic sensor for environmental monitoring. Opt. Express.

[12] Lewicki R., Wysocki G., Kosterev A.A., Tittel F.K. (2007). QEPAS based detection of broad-band absorbing molecules using a widely tunable, cw quantum cascade laser at 8.4 µm. Opt. Express.

[13] Kosterev A.A., Dong L., Thomazy D., Tittel F.K., Overby S. (2010). QEPAS for chemical analysis of multi-component gas mixtures. Appl. Phys. B.

[14] Zampolli S., Mengali S., Liberatore N., Elmi I., Masini L., Sanmartin M., Viola R. (2020). A MEMS-Enabled Deployable Trace Chemical Sensor Based on Fast Gas-Chromatography and Quartz Enhanced Photoacoustic Spectroscopy. Sensors.

[15] Patimisco P., Sampaolo A., Dong L., Giglio M., Scamarcio G., Tittel F.K., Spagnolo V. (2016). Analysis of the electro-elastic properties of custom quartz tuning forks for optoacoustic gas sensing. Sens. Actuators B Chem..

[16] Viola R., Liberatore N., Luciani D., Mengali S. (2016). Quartz enhanced photoacoustic spectroscopy for detection of improvised explosive devices and precursors. Adv. Opt. Technol..

[17] Köhring M., Pohlkötter A., Willer U., Angelmahr M., Schade W. (2011). Tuning fork enhanced interferometric photoacoustic spectroscopy: A new method for trace gas analysis. Appl. Phys. B.

[18] He Y., Ma Y., Tong Y., Yu X., Tittel F.K. (2018). HCN ppt-level detection based on a QEPAS sensor with amplified laser and a miniaturized 3D-printed photoacoustic detection channel. Opt. Express.

[19] Dello Russo S., Zhou S., Zifarelli A., Patimisco P., Sampaolo A., Giglio M., Iannuzzi D., Spagnolo V. (2020). Photoacoustic spectroscopy for gas sensing: A comparison between piezoelectric and interferometric readout in custom quartz tuning forks. Photoacoustics.

[20] Dong L., Kosterev A.A., Thomazy D., Tittel F.K. (2010). QEPAS spectrophones: Design, optimization, and performance. Appl. Phys. B.

[21] Patimisco P., Sampaolo A., Zheng H., Dong L., Tittel F.K., Spagnolo V. (2017). Quartz–enhanced photoacoustic spectrophones exploiting custom tuning forks: A review. Adv. Phys..

[22] Sharpe S.W., Sams R.L., Johnson T.J. The PNNL Quantitative IR Database for Infrared Remote Sensing and Hyperspectral Imaging. Proceedings of the 31st Applied Imagery Pattern Recognition Workshop.

